# Ovarian fibrothecoma in a 15-year-old girl: A rare case report

**DOI:** 10.1016/j.radcr.2025.04.011

**Published:** 2025-05-05

**Authors:** Abourak Chaimae, El Haddad Siham, Bouljrouf Jaoud, Kisra Mounir, Oukassem Siham, Guennouni Asmae, Bahha Soukaina, Belkouchi Lina, Allali Nazik, Chat Latifa

**Affiliations:** a Department of Radiology, Mother-Child, Faculty of Medicine and Pharmacy of Rabat, Children's Hospital, Ibn Sina University Hospital, Mohammed V University, Rabat, Morocco; b Department of Pediatric Surgery, Mother-Child Hospital, Faculty of Medicine and Pharmacy of Rabat, Children's Hospital, Ibn Sina University Hospital, Mohammed V University, Rabat, Morocco

**Keywords:** Ovarian fibrothecoma, Adolescent gynecology, Magnetic resonance imaging (MRI), Differential diagnosis

## Abstract

Ovarian fibrothecomas are rare benign tumors typically found in middle-aged women. This report presents a case of a 15-year-old girl with an irregular menstrual cycle and a left lateral uterine mass, ultimately diagnosed as an ovarian fibrothecoma. This case highlights the diagnostic challenges posed by fibrothecomas in younger patients, as they may mimic malignant ovarian tumors on imaging. Magnetic resonance imaging (MRI) findings, surgical intervention, and pathological examination confirmed the diagnosis. This case emphasizes the importance of accurate imaging and multidisciplinary approaches in managing such ovarian masses in adolescents.

## Introduction

Ovarian fibrothecomas are rare benign sex cord-stromal tumors that account for less than 1% of ovarian neoplasms. They are more commonly diagnosed in postmenopausal women, with a peak incidence in the fifth to sixth decades of life [1]. The occurrence of such tumors in adolescents is exceedingly rare, and their clinical and radiological features can mimic more aggressive pathology, such as germ cell or epithelial ovarian tumors [2]. This report discusses a case of ovarian fibrothecoma in a 15-year-old girl, underscoring the importance of distinguishing benign tumors from malignancies in young patients. The diagnostic challenges presented by fibrothecomas in this age group necessitate a careful approach, as imaging characteristics can often overlap with those of malignant neoplasms [3].

## Observation

This is a 15-year-old girl**,** with no notable personal or family history. She had her menarche 1 year ago with irregular cycles, which prompted her to consult a general practitioner. The doctor discovered a left lateral uterine mass in a context of preserved general condition, and the clinical examination was unremarkable. A pelvic ultrasound was performed, revealing a hypoplastic uterus with a left lateral uterine mass, likely of ovarian origin.

**Fig. 1:**
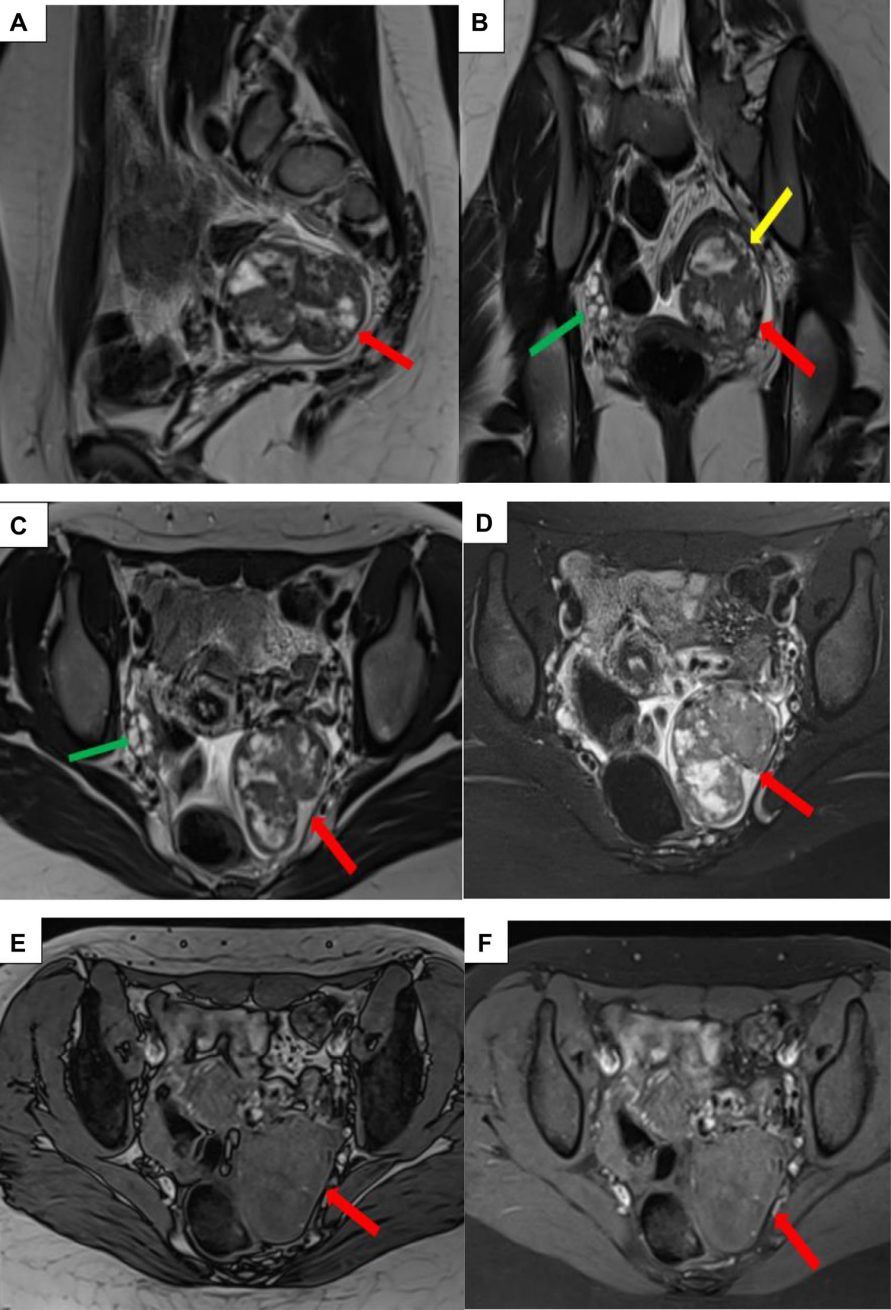

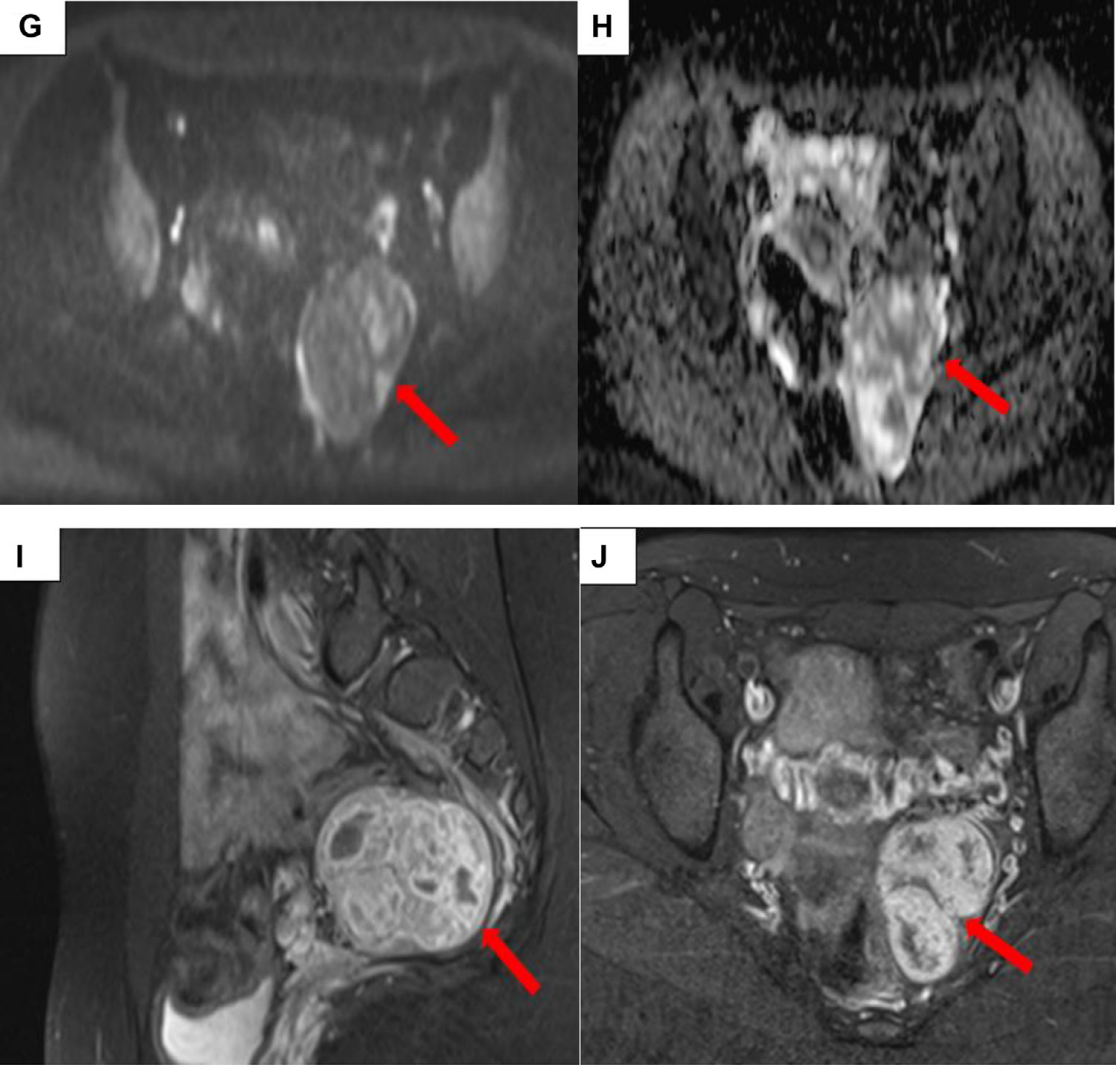
Pelvic MRI: Sagittal T2-weighted (A), coronal T2-weighted (B), axial T2-weighted (C), axial T2-weighted with fat saturation (D), axial T1-weighted (E), axial T1-weighted with fat saturation (F), diffusion-weighted imaging (G), ADC map (H), coronal and axial contrast-enhanced T1-weighted images with fat saturation (I and J). A left lateral uterine mass (red arrow) demonstrating the claw sign with the left ovary (yellow arrow), well-defined, with regular contours and surrounded by a thin capsule. The lesion is heterogeneous, with an intermediate T2-weighted signal, isointense on T1-weighted images, and does not suppress on fat-saturated sequences (confirming the absence of fat components). It exhibits mild hyperintensity on diffusion-weighted imaging with a slightly low ADC, strong enhancement after gadolinium administration, and a minor cystic component with T2 hyperintensity. The right ovary is follicular, with normal size and morphology (green arrow).

A complementary pelvic MRI was performed to further characterize the mass and confirm its origin. It revealed a well-defined left lateral uterine mass with regular contours, demonstrating the claw sign with the left ovary. The lesion appeared heterogeneous, with an intermediate T2-weighted signal, isointense on T1-weighted images, and did not suppress on fat-saturated sequences (confirming the absence of fatty components). A mild diffusion hyperintensity with a slightly decreased ADC was noted, along with strong enhancement after gadolinium injection. Additionally, a minor cystic component with T2 hyperintensity was identified, measuring 58 × 49 × 44 mm (Fig. 1).

Topographically, this mass was separated from the anterior rectal wall by a thin fat plane, with preservation of the uterine body, cervix, and vaginal canal anteriorly. No fatty component or calcifications were detectable, particularly on the complementary CT scan. Associated with this were a few perilesional lymph nodes, the largest measuring 8 mm, along with a small amount of pelvic fluid. The diagnosis of a malignant ovarian mass, initially suggesting a malignant germ cell tumor, was made.

**Fig. 2:**
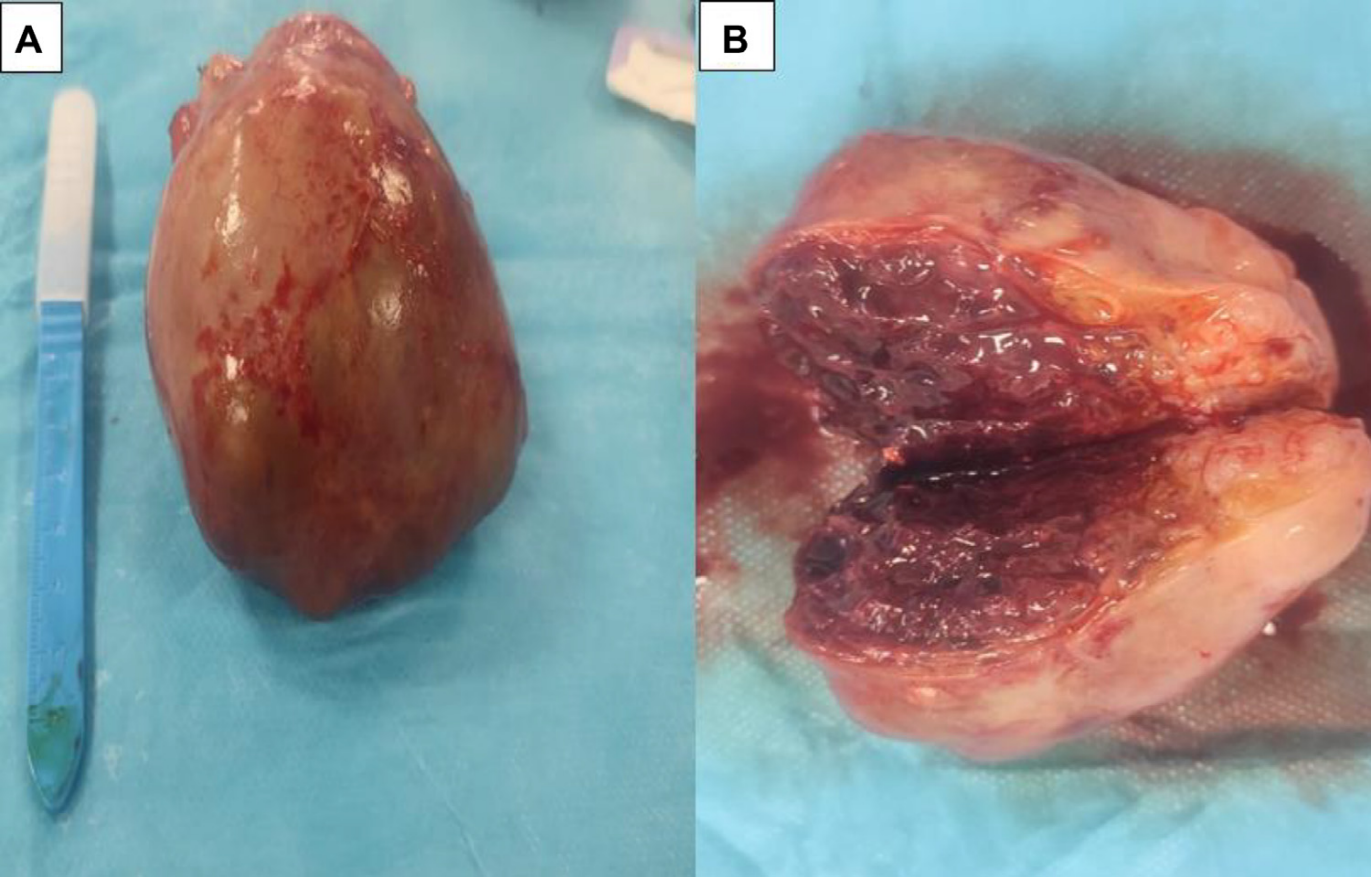
Intraoperative images of the left lateral uterine mass (A, B). The macroscopic specimen appears as a well-defined, encapsulated ovarian mass with a firm to hard consistency. On sectioning, the surface is homogeneous, grayish-white or yellowish, with occasional fibrous or edematous areas (A). Cystic foci may be observed but are rare. There are generally no signs of significant necrosis or hemorrhage. The internal architecture shows a lobulated appearance, often consistent with the fibrous nature of the tumor (B).

The biological work-up was normal, with β-HCG at 2 mIU/mL, alpha-FP at 2.3 mIU/mL, and CA 125 at 4.1 mIU/mL. The decision for surgical removal of the mass was made during a multidisciplinary meeting. The macroscopic examination of the surgical specimen was in favor of a fibrothecoma (Fig. 2), confirmed by microscopic pathological examination. Postoperative outcomes were unremarkable.

## Discussion

Ovarian fibrothecomas are a subtype of sex cord-stromal tumors characterized by their combined fibrous and thecal components. These tumors are generally benign and rare, particularly in adolescents, making this case of a 15-year-old patient remarkable. Typically, fibrothecomas present in women in their forties and sixties, with an average age of diagnosis around 50 years [1]. The occurrence of such a tumor in a young patient raises diagnostic challenges, as these tumors can mimic more aggressive ovarian malignancies, particularly on imaging [2].

In this case, the patient presented with irregular menstrual cycles, a common but nonspecific symptom that prompted further evaluation. The pelvic ultrasound initially suggested a mass of ovarian origin, consistent with most ovarian fibrothecomas, which often appear as solid adnexal masses [3]. However, ultrasound characteristics alone are insufficient to establish a definitive diagnosis, especially to differentiate benign ovarian tumors from malignant ones.

Magnetic resonance imaging (MRI) was essential for further characterizing the mass. The MRI findings in this case were consistent with the typical characteristics of fibrothecomas, which include intermediate signal intensity on T2-weighted images, isointensity on T1-weighted images, and strong enhancement postcontrast due to their rich vascular stroma [4]. The absence of fat components and calcifications, as confirmed by the complementary CT scan, helped exclude other differential diagnoses such as teratomas [5]. However, the minor cystic component and the presence of perilesional lymphadenopathy raised concerns about a potentially malignant process, particularly in a young patient.

Despite the concerning imaging characteristics, the normal tumor marker profile (β-HCG, AFP, and CA 125) decreased the likelihood of germ cell tumors or epithelial ovarian cancers [6]. Tumor markers are often useful for distinguishing between different types of ovarian neoplasms, especially in young women where germ cell tumors are more common [7]. Nonetheless, the imaging results and clinical presentation justified surgical intervention to confirm the diagnosis.

Surgical resection remains the gold standard for diagnosis and treatment. In this case, laparoscopic excision provided both treatment and diagnostic confirmation. Histopathological analysis confirmed the tumor as a fibrothecoma, underscoring the importance of tissue diagnosis in such cases [8]. Fibrothecomas are typically well-encapsulated and have a favorable prognosis following surgical removal, with recurrence being rare [9].

Management of ovarian masses in adolescents poses unique challenges, as the differential diagnosis is broad, ranging from functional cysts to malignancies. The rarity of fibrothecomas in this age group makes early and accurate diagnosis crucial to avoid unnecessary aggressive treatment. Conservative surgical approaches, such as laparoscopy, are preferred to preserve ovarian function, particularly in young patients [2].

This case contributes to the limited literature on ovarian fibrothecomas in adolescents and highlights the need for a multidisciplinary approach, including radiological, surgical, and pathological expertise. It also underscores the utility of MRI in preoperative workup, allowing for detailed characterization of the mass and guiding surgical approach . However, as this case illustrates, imaging characteristics may be nonspecific, and the definitive diagnosis often relies on histopathological evaluation.

## Conclusion

This case highlights the importance of considering benign ovarian tumors, such as fibrothecomas, in the differential diagnosis of ovarian masses in adolescents. MRI provides valuable information, but definitive diagnosis requires histopathological confirmation. A multidisciplinary approach, including input from radiologists, surgeons, and pathologists, is essential for the optimal management of such cases.

## Ethics approval

Our institution does not require ethical approval for reporting individual cases or case series.

## Patient consent

Written informed consent was obtained from the patient(s) for their anonymized information to be published in this article.
